# AKR1B1-Induced Epithelial–Mesenchymal Transition Mediated by RAGE-Oxidative Stress in Diabetic Cataract Lens

**DOI:** 10.3390/antiox9040273

**Published:** 2020-03-25

**Authors:** Tsung-Tien Wu, Ying-Ying Chen, Hui-Yu Chang, Ya-Hsin Kung, Ching-Jiunn Tseng, Pei-Wen Cheng

**Affiliations:** 1 Department of Ophthalmology, Kaohsiung Veterans General Hospital, Kaohsiung 81362, Taiwan; ttwu@vghks.gov.tw (T.-T.W.); yychen@vghks.gov.tw (Y.-Y.C.); yhkung@vghks.gov.tw (Y.-H.K.); 2 Faculty of Medicine, National Yang-Ming University, Taipei 11221, Taiwan; 3 Department of Medical Education and Research, Kaohsiung Veterans General Hospital, Kaohsiung 81362, Taiwan; abc76740960@hotmail.com (H.-Y.C.); cjtseng@vghks.gov.tw (C.-J.T.); 4 Department of Biomedical Science, National Sun Yat-Sen University, Kaohsiung 80424, Taiwan

**Keywords:** AKR1B1, diabetic cataracts, reactive oxygen species (ROS), AMPK, epithelial–mesenchymal transition (EMT)

## Abstract

Purpose: Cataracts are a major cause of visual acuity deterioration in diabetes mellitus (DM) in developed and developing countries. Studies have demonstrated that overproduction of AKR1B1 and receptor for advanced glycation end products (RAGE) plays a major role in the pathogenesis of diabetic cataracts, but it is unclear whether the prevalence of diabetic cataracts is related to epithelial–mesenchymal transition (EMT) in lens epithelial cells. This study aimed to analyze the role of EMT in cataract formation of DM patients. Methods: Immunofluorescence and immunohistochemistry assays were used to estimate AKR1B1, RAGE, AMPK, and EMT levels in epithelial human lens of DM or non-DM cataracts. Results: Immunohistochemical staining demonstrated that pathologic phases and N-cadherin expression levels were significantly higher in epithelial human lens of DM (+) compared to DM (−) cataracts. Immunofluorescent staining showed that AKR1B1 and RAGE were significantly higher in epithelial human lens of DM (+) compared to DM (−) cataracts. Interestingly, acetyl superoxide dismutase 2 (AcSOD2) levels were significantly higher in DM patients’ lens epithelial cells (LECs), whereas AMPKT172 phosphorylation was significantly increased in non-DM patients. This indicates that AMPKT172 might be related to superoxide reduction and diabetic cataract formation. Conclusions: Our results suggest that AKR1B1 overexpression can decrease AMPK activation, thereby increasing AcSOD2 and RAGE-induced EMT in epithelial human lens of DM cataracts. These novel findings suggest that AKR inhibitors may be candidates for the pharmacological prevention of cataracts in patients with DM.

## 1. Introduction

The International Diabetes Federation estimates that diabetes mellitus (DM) population will reach 439 million people in 2030. An aging population and extended patient life expectancy might cause DM patients to exceed 33% by 2050 [1]. Hyperglycemia is a risk factor for numerous diabetic complications, including diabetic cataracts (DCs) development. DCs are characterized by lens opacity and are one of the earliest secondary complications of DM [2,3]. Chronic hyperglycemia leads to osmotic damage, oxidative stress, and non-enzymatic glycation processes in the optical, which are primary drivers for DCs development [2].

The mechanism of cataract formation is not well understood. Recent studies support the view that there is a close relationship between cataracts and epithelial–mesenchymal transition (EMT) [4]. During the EMT process, epithelial cells lose several of their epithelium-specific characteristics, including E-cadherin expression decrease, typical mesenchymal cell properties’ enhancement, such as N-cadherin increased expression. Many factors can lead to EMT, including transforming growth factor-β (TGF-β). Raghavan et al. reported that advanced glycation end products (AGEs) in the human lens capsule promoted TGFβ2-mediated lens epithelial cell EMT [5]. Previous studies showed that dietary sugars and receptor for advanced glycation end products (RAGE) activation mediate AGEs generation, which eventually leads to metabolic syndrome [6,7]. Additionally, AGEs and their receptor RAGE may directly induce superoxide through aldose reductase (AR) and/or other mechanisms. Furthermore, diabetic lenses are more susceptible to oxidative stress compared to normal lenses that results from dysfunctional antioxidant capability. Superoxide dismutase (SOD) is a predominant antioxidant enzyme in the lens that degrades superoxide radicals (O^2−^) into H_2_O_2_ and oxygen. SOD also protects the lens against cataract development in DM models [8,9]. As a consequence, decreased activity of antioxidant enzymes and reducing substances, excessive AR activation, and AGE accumulation are risk factors for DC occurrence [10,11].

AR (*AKR1B1* in humans) is an NADPH-dependent aldo–keto reductase (ALR), a well-studied catalyst of glucose conversion to sorbitol in the polyol pathway, and is believed to be a key player in the cataract development mechanism [12]. Chang et al. revealed that *AKR1B1* overexpression in the lens developed anterior subcapsular cataracts in vivo, even without diabetes and hyperglycemia [13]. However, *ALR2* expression in the lens varies among different animal species, whereby animals with *ALR2* deficiency are relatively more resistant to diabetic cataract formation [14]. Genetic polymorphisms linked to the human *AKR1B1* gene are associated with higher tissue levels of ALR2 and the development of advanced diabetic retinopathy [15]. ALR2 inhibitors effectively suppress lens epithelial cell proliferation and their transition to mesenchymal cells in the EMT process [13].

Previously, it was shown that sorbitol accumulation caused osmotic stress in the lens that led to epithelial cell apoptosis and cataract formation [16]. In fact, rapid glycemic control promotes a hypoxic environment that reduces protective enzymes and increases oxidative radicals. This study demonstrates that osmoregulation impairment leads to the lens being susceptible to AR-mediated osmotic stress and eventually, cataract formation. Overproduction of AKR1B1 and RAGE also plays a major role in diabetic cataract development. However, it is not yet known whether diabetic cataracts are related to lens epithelial cell EMT. In the present study of diabetic eye disease, we hypothesize that EMT in DM cataracts occurs through AKR1B1-enhanced AGE, and reactive oxygen species (ROS) generation influences the cataract development in DM. Based on our data, AKR1B1 overexpression may diminish AMPK activation, and increase AcSOD2- and RAGE-induced EMT in the lens epithelial cells (LECs) of diabetic cataracts.

## 2. Materials and Methods

### 2.1. Ethics Statement

Study protocols were independently reviewed and approved by the Institutional Review Board at the Kaohsiung Veterans General Hospital (Kaohsiung, Taiwan; IRB number: VGHKS17-CT5-10; VGHKS18-CT4-22). The study protocol was approved by the hospital’s institutional review board. After receiving a full explanation of the surgical procedures and possible complications, informed consent was provided by all patients. Patients were selected based on clinically observable grade 2 or 3 nuclear cataracts, as measured by the Lens Opacities Classification System III [17]. The central flap of anterior lens capsular consisted of a single layer of lens epithelium with apices directed inward, a basal laminar which forms the lens capsule. When patients had undergone phacoemulsification, the central flap of the anterior lens capsule was taken from the patients. The anterior region of the lens capsule from the patient was obtained for immunofluorescence staining. All cataract surgeries were performed by the surgeons Ying-Ying Chen, Tsung-Tien Wu, or Ya-Hsin Kung. Patients were classified into two groups: patients without DM (Group 1) and patients with DM but without diabetic retinopathy (Group 2).

### 2.2. Reagents

Primary antibodies for RAGE and nitrotyrosine (sc-8230 and sc-32757, respectively, Santa Cruz, CA, USA), E- and N-cadherin (610182 and 610920, respectively, BD Biosciences, San Jose, CA, USA), AKR1B1, MMP9, vimentin (GTX113381, GTX100458, and GTX100619, respectively, Genetex, Irvine, CA, USA), SOD2 (acetyl K68) (ab137037, Abcam, Eugene, OR, USA), and Phosph-AMPK α1,2^T172^ (44-1150G, Thermo Fisher Scientific, Eugene, OR, USA) were used for immunofluorescence staining.

### 2.3. Immunohistochemical Procedures

**Figure 1:**
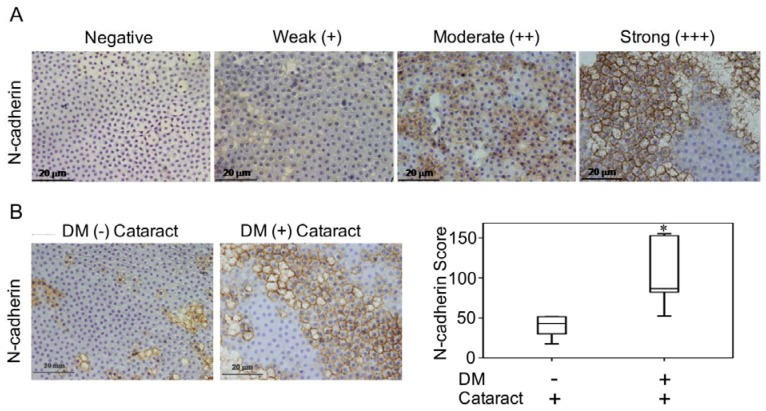
N-cadherin in diabetes mellitus (DM) (+) and DM (−) cataract patients. (**A**) Representative photomicrographs of cataract samples with N-cadherin labeled to score negative (−), weak (+), moderate (++), and strong (+++) expressions and the pathological phase of the cataracts (phase 1-4). (**B**) In DM (+) cataract patients, N-cadherin expression levels were higher than those in DM (−) cataract patients (*p* = 0.040). (**C**) Box plot of N-cadherin expression levels in DM (+) and DM (−) cataract patients. The presented values are the mean ± SEM (*n* = 10 per group, separate experimental groups in each figure). * *p* < 0.05.

Lenses were incubated with N-cadherin (BD Biosciences, San Jose, CA, USA) overnight at 4 °C. Color was developed with a solution of 0.03% diaminobenzidine, and sections were counterstained with hematoxylin. Immune reactivity was scored using a semi-quantitative approach based on staining intensity and staining percentage. Staining intensity was scored as 0 when no positive cells were identified, 1 as being weak, 2 as moderate, and 3 as having strong signal (Figure 1A). Staining percentages were multiplied by intensity scores to obtain final scores.

### 2.4. Immunofluorescence Staining

Samples were permeabilized in 0.5% Triton X-100 and blocked with 10% normal horse serum. Next, specimens were incubated in primary antibodies in the presence of 10% normal horse serum at 4 °C overnight. After PBS washing, sections were incubated with Alexa Fluor 488- or 588 (Invitrogen). The immunofluorescence technique was performed in accordance with approved protocols [18], and negative controls performed to check binding specificity. Briefly, marginal square regions were drawn around the signal of interest, and the integrated intensity values were recorded. Based on the marked margin, intensities within these squares were calculated. The sections were analyzed using fluorescence microscopy and Zeiss LSM Image software (Carl Zeiss Micro Imaging, Jena, Germany). 

### 2.5. Statistical Analyses

Student’s *t*- and Mann–Whitney U tests, ANOVAs, and Kruskal–Wallis one-way ANOVAs were used to evaluate the relationships between DM and non-DM cataract patients. All statistical analyses were conducted using SPSS, version 20.0 (SPSS Inc, Chicago, IL, USA). A *p* < 0.05 (two-sided) was considered significant. Data are represented as means ± SEMs. 

## 3. Results

### 3.1. Demographic Characteristics

This study included cataract patients with (*N* = 35) and without (*N* = 35) DM. Refraction and axial length were assessed in 15 patients in the DM group and 15 patients in the non-DM group, while the axial length was measured in 18 DM (+) and 12 DM (−) patients. The average age of the DM (−) cataract patients was 62 ± 9.5 years. The average age of the DM (+) cataract patients was 62.3 ± 7 years (*p* = 0.2). In total, 15 males and 15 females were included in the DM (+) group, while 18 males and 12 females were included in the DM (−) group.

### 3.2. N-Cadherin May Contribute to the Formation of Diabetic Cataracts

Previous work demonstrated that LECs that undergo EMT contribute to cataract formation [19,20]. In this study, we investigated the pathologic phases and N-cadherin expression levels of cataracts in patients with and without DM (Figure 1A). We found that N-cadherin expression in DM (+) cataract tissues was significantly higher than that in DM (−) patients (*p* = 0.040, Figure 1B,C). This suggests that N-cadherin, a mesenchymal marker, potentially plays a role in DM (+) cataract patients.

### 3.3. Activated AKR1B1, enhancedRAGE Production Were Involved in DM (+) Cataract Pathogenesis of LECs

**Figure 2:**
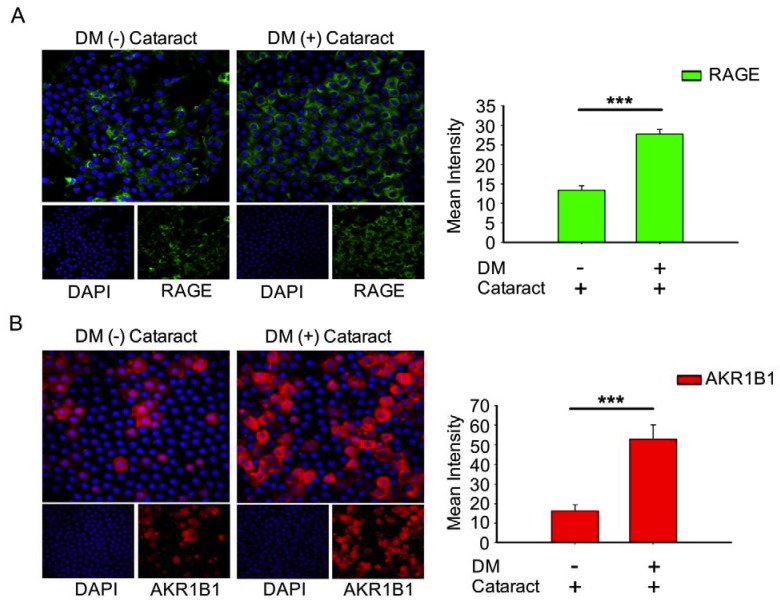
Expression of receptor for advanced glycation end products (RAGE) and AKR1B1 were higher in the lens in DM (+) cataract patients than in DM (−) cataract patients. (**A**) Representative green fluorescence images of green RAGE-positive and (**B**) red AKR1B1-positive cells in human lens epithelial sections from DM (+) or DM (−) cataracts. Cell nuclei were counterstained with DAPI (blue). Values are presented as means ± SEMs (*n* = 10 per group, separate experimental groups in each figure). *** *p* < 0.001.

AKR1B1 and RAGE both contribute to diabetic eye disease [12,21,22]. Here, we evaluated AKR1B1 and RAGE expressions in DM (+) and DM (−) cataract patients. Both proteins were expressed at significantly higher levels in the DM (+) cataract group than in the control group (both *p* < 0.001, Figure 2). These observations imply that AKR1B1 and RAGE are involved in the pathogenesis of diabetic cataracts.

### 3.4. Inhibition of AMP-Activated Protein Kinase (AMPK) Increased ROS Production and Acetylation of SOD2 in the LECs of DM (+) Patients

**Figure 3:**
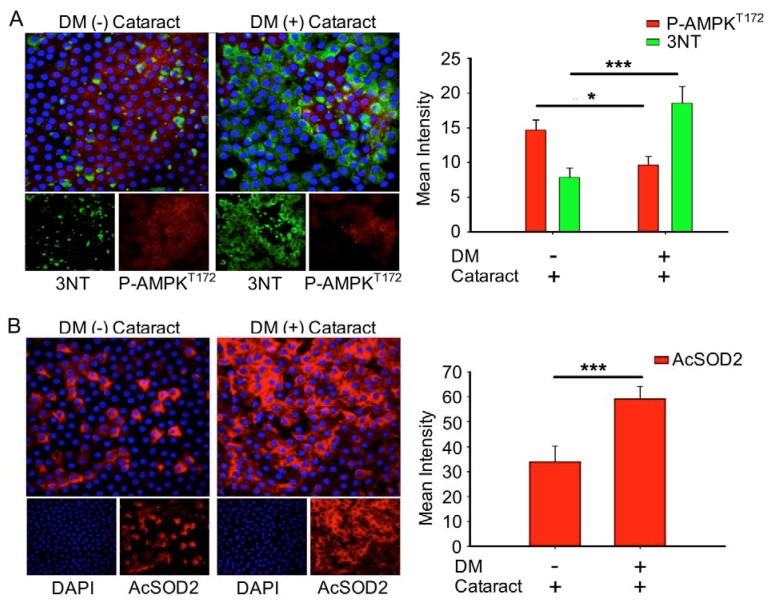
DM (+) cataract patients had increased energy imbalance, increased reactive oxygen species (ROS) products, and lower superoxide dismutase 2 SOD2 activity than DM (−) cataract patients. (**A**) Representative photomicrographs of p-AMPK^T172^ (red) and 3-nitrotyrosine (3NT, green), and (**B**) of acetyl SOD2 (AcSOD2, red). DAPI (blue) was used to label cell nuclei. Values are presented as means ± SEMs (*n* = 10 per group, separate experimental groups in each figure). * *p* < 0.05 and *** *p* < 0.001.

Misra and Chakrabarti found that enhanced AMPK activity reduced the severity of type two diabetes [23], and Tsubota et al. showed that lens inflammation downregulated the AMPK pathway in diabetic mice [24]. In addition, oxidative stress plays an important role in the pathogenesis of metabolic syndrome [25]. These findings indicate that AMPK activity and superoxide accumulation are crucial for diabetic patients. Here, we investigated the status of p-AMPK^T172^ and superoxide in cataract patients with and without diabetes. We demonstrated that DM (+) cataract patients had decreased levels of p-AMPK^T172^-induced 3-nitrotyrosine, an ROS product, compared to DM (−) cataract patients (*p* = 0.014 and *p* < 0.001, respectively) (Figure 3A). We also examined the effects of DM on ROS product supersession efficiency by measuring AcSOD2 expression levels. By immunofluorescence, we found that AcSOD2 expression was significantly higher in the DM (+) compared to the DM (−) group (*p* < 0.001, Figure 3B).

### 3.5. EMT Was Associated with Cataracts, the Mesenchymal Cell Marker N-Cadherin, and MMP9 in DM (+) Cataract Patients

**Figure 4:**
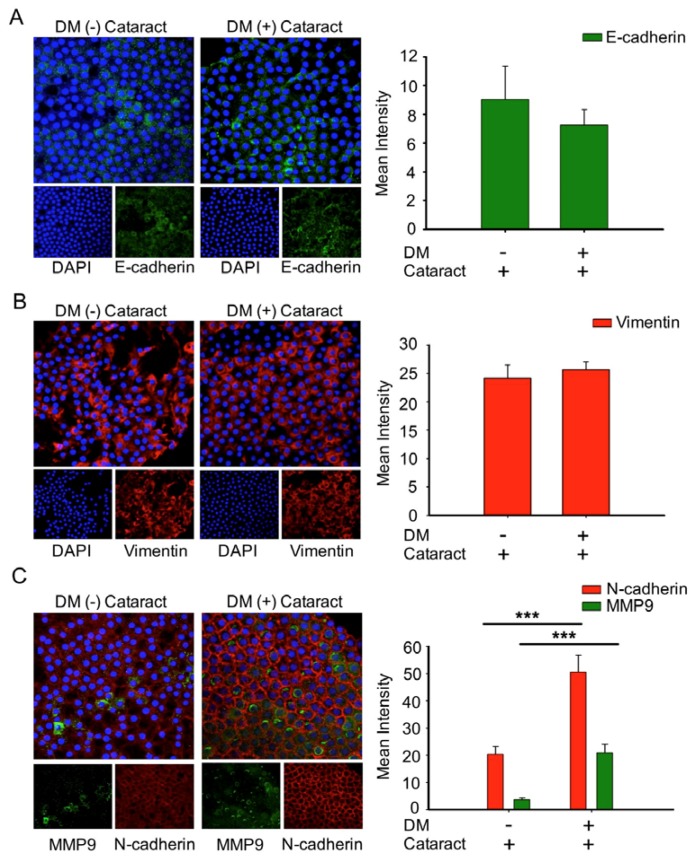
Cataracts induced epithelial–mesenchymal transition (EMT) progression with significantly elevated N-cadherin and MMP9 expression in DM (+) patients. (**A**) Representative green fluorescence images of E-cadherin and (**B**) red fluorescence images of vimentin-positive cells in human lens epithelial sections from DM (+) or DM (−) cataracts. (**C**) MMP9 (green) and N-cadherin (red) were both upregulated in DM (+) cataract patients compared to DM (−) cataract patients. Lens cell nuclei were counterstained with DAPI (blue). Presented values are means ± SEMs (*n* = 10 per group, separate experimental groups in each figure). *** *p* < 0.001.

To determine the role of EMT in cataract formation, EMT markers of cataracts in DM and non-DM patients were analyzed (Figure 4). We observed that the expression of E-cadherin, an epithelial marker, was lower in DM (+) cataract tissues compared to DM (−) patients (*p* = 0.06, Figure 4A). We found that vimentin expression in DM (+) cataract tissues showed no significance compared to that in DM (−) patients (25.67 ± 1.4 vs. 24.2 ± 2.3; *p* = 0.07, Figure 4B). However, the mesenchymal marker N-cadherin, and matrix metalloproteinases 9 were increased significantly in DM (+) cataract patients compared to DM (−) cataract patients (Figure 4C), showing that the mesenchymal marker N-cadherin may play an important role in DM (+) cataract formation.

## 4. Discussion

Diabetes likely affects more than 400 million adults worldwide [26], and cataracts are a frequent ocular complication in these patients. Furthermore, the relationship between cortical cataracts or fasting blood glucose and diabetes has been well-established from numerous population-based studies [27]. However, the mechanisms underlying cataract development in diabetic patients are not well understood.

Growing evidence indicates the importance of both elevated blood glucose and genetic factors in diabetic cataract development. Reports have associated variations in the *AKR1B1* gene with diabetic retinopathy increasing diabetic retinopathy risk [28]. Few studies examined the relationship between *AKR1B*, glucose levels, and cataract formation [29]. The association we found in this study between elevated fasting blood glucose and cataracts is consistent with previous reports [27].

Tan et al. further demonstrated the major role of elevated blood glucose in the development of cataracts [30]. The mechanisms underlying cataract development that accompany blood glucose elevation and lens protein glycosylation are related to subsequent opacity and oxidation [31], formation of ROS, and sorbitol accumulation through AR conversion of glucose [32]. AGEs mediates superoxide generation to activation of NADPH oxidase, which leads to altered gene expression via RAGE [33]. Recently, AGEs were found to be a key player in lens degeneration. As a result, preventative approaches utilizing low AGE foods may be beneficial for counteracting cataract formation [34].

Additional work has proposed mechanisms for diabetic retinopathy and potential targets for prevention. Kumamoto et al. reported that elevated erythrocyte ALR2 strongly correlated with decreased LEC density, diabetic retinopathy, and higher HbA1c [35]. These authors concluded that the polyol pathway might be associated with epithelial cell reduction in the DM patients’ eyes through ALR2. Consistently, we observed that increased AKR1B1 was significantly correlated with decreased LEC density, which enhanced EMT in DM (+) cataract patients (Figure 1). Interestingly, AKR1B1 and RAGE levels were only significantly elevated in the LECs of DM (+) patients in the present study (Figure 2). Our previous work similarly demonstrated that sodium-dependent glucose cotransporter 2 (SGLT2) inhibitors enabled glycemic control in type 2 diabetes mellitus (T2DM) patients, by downregulating RAGE-induced NADPH oxidase expression in LECs via the inactivation of GLUTs and reduced ROS generation [9]. Wang S. et al. reported that AMPKα2 deletion enhanced NADPH oxidase subunit expression and increased oxidative stress in vascular endothelial cells [36]. Misra and Chakrabarti found that enhanced AMPK activity reduced the severity of type two diabetes [23], and Tsubota et al. showed that lens inflammation downregulated the AMPK pathway in diabetic mice [24]. These findings indicate that AMPK activity and superoxide accumulation are crucial for diabetic patients. Here, we demonstrated that 3-NT and AcSOD2 levels in LEC sections were significantly higher, while p-AMPK^T172^ levels were significantly lower in DM (+) cataract patients (Figure 3). These results indicate that AKR1B1 may be required for RAGE-induced superoxide generation and diabetic cataract formation. The AKR superfamily of NADP(H)-dependent aldehyde and ketone reductases has already been explored extensively as drug targets in pharmaceutical and clinical trials. For example, AKR inhibitors, such as tolrestat and epalrestat, were developed to target AKR1B1 for treating type two diabetes complications [37]. AKR1B1 inhibitors may be promising pharmacological agents that prevent cataracts in diabetic patients.

After breaching the blood–aqueous barrier during cataract surgery, active TGFβ increases with subsequent inflammatory response, which increases latent TGFβ activators, such as MMPs, thrombospondin, and αV integrins [38]. This leads to sustained TGFβ activation, which drives the fibrotic events that lead to posterior capsule opacification (PCO). Current evidence supports the view that there is a close relationship between cataracts and EMT [20]. Du et al. reported that oxidative stress increases AR activation and AGE production, driving EMT in the early stages of diabetic cataract formation [20]. Furthermore, Korol at al. reported a contribution of MMPs in EMT to fibrotic disease in mouse models of ASC [39]. The present study further reveals that N-cadherin and MMP9 levels are significantly elevated in DM (+) cataract patients. This study also finds that vimentin levels in LEC sections were both higher in cataract patients with and without DM (Figure 4).

## 5. Conclusions

**Figure 5:**
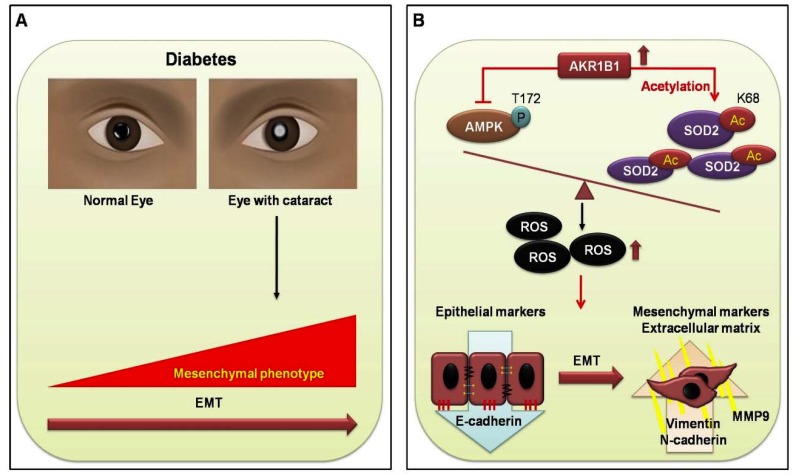
Proposed signal transduction cascades in DM (+) and DM (−) cataract patients. (**A**) Current evidence supports the view that cataracts are a major cause of visual acuity deterioration in DM; however, that relationship is still unclear. In the present study of diabetic eye disease, we hypothesize that EMT in DM cataracts occurs through AKR1B1-enhanced advanced glycation end products (AGE), and ROS generation influences the cataract development in DM. (**B**) AKR1B1 overexpression inhibited AMPK activation, thereby increasing AcSOD2 and ROS-induced EMT in the lens epithelial cells LECs of diabetic cataracts. This suggests a potentially important mechanism for the pathogenesis of diabetic cataracts.

In conclusion, we found that EMT in the DM cataract occurs through AKR1B1-enhanced AGE and ROS generation in LECs from DM (+) cataract patients. Furthermore, AKR1B1 overexpression inhibited AMPK activation, thereby increasing AcSOD2 and RAGE-induced EMT in the LECs of diabetic cataracts. This suggests a potentially important mechanism for the pathogenesis of diabetic cataracts (Figure 5). AKR inhibitors have been developed to target AKR1B1 for the treatment of complications that accompany T2DM and could be delivered by eye drops to treat diabetic cataracts, though further work is necessary to determine optimal delivery routes. Finally, manipulation of this pharmacological cascade may prevent cataracts in patients with diabetes, which will benefit many cataract patients.

## References

[1] Boyle J.P., Thompson T.J., Gregg E.W., Barker L.E., Williamson D.F. (2010). Projection of the year 2050 burden of diabetes in the us adult population: Dynamic modeling of incidence, mortality, and prediabetes prevalence. Popul. Health Metr..

[2] Pollreisz A., Schmidt-Erfurth U. (2010). Diabetic cataract-pathogenesis, epidemiology and treatment. J. Ophthalmol..

[3] Albulescu R., Zolog I. (2011). Specific aspects in diabetic cataract. Oftalmologia.

[4] Wernecke L., Keckeis S., Reichhart N., Strauss O., Salchow D.J. (2018). Epithelial-mesenchymal transdifferentiation in pediatric lens epithelial cells. Investig. Ophthalmol. Vis. Sci..

[5] Raghavan C.T., Nagaraj R.H. (2016). Age-rage interaction in the tgfbeta2-mediated epithelial to mesenchymal transition of human lens epithelial cells. Glycoconj. J..

[6] Guglielmotto M., Aragno M., Tamagno E., Vercellinatto I., Visentin S., Medana C., Catalano M.G., Smith M.A., Perry G., Danni O. (2012). Ages/rage complex upregulates bace1 via nf-kappab pathway activation. Neurobiol. Aging.

[7] Miller A., Adeli K. (2008). Dietary fructose and the metabolic syndrome. Curr. Opin. Gastroenterol..

[8] Olofsson E.M., Marklund S.L., Behndig A. (2009). Enhanced diabetes-induced cataract in copper-zinc superoxide dismutase-null mice. Investig. Ophthalmol. Vis. Sci..

[9] Chen Y.Y., Wu T.T., Ho C.Y., Yeh T.C., Sun G.C., Kung Y.H., Wong T.Y., Tseng C.J., Cheng P.W. (2019). Dapagliflozin prevents nox- and sglt2-dependent oxidative stress in lens cells exposed to fructose-induced diabetes mellitus. Int. J. Mol. Sci..

[10] Kandarakis S.A., Piperi C., Topouzis F., Papavassiliou A.G. (2014). Emerging role of advanced glycation-end products (ages) in the pathobiology of eye diseases. Prog. Retin. Eye Res..

[11] Sadi G., Eryilmaz N., Tutuncuoglu E., Cingir S., Guray T. (2012). Changes in expression profiles of antioxidant enzymes in diabetic rat kidneys. Diabetes Metab. Res. Rev..

[12] Snow A., Shieh B., Chang K.C., Pal A., Lenhart P., Ammar D., Ruzycki P., Palla S., Reddy G.B., Petrash J.M. (2015). Aldose reductase expression as a risk factor for cataract. Chem. Biol. Interact..

[13] Zablocki G.J., Ruzycki P.A., Overturf M.A., Palla S., Reddy G.B., Petrash J.M. (2011). Aldose reductase-mediated induction of epithelium-to-mesenchymal transition (emt) in lens. Chem. Biol. Interact..

[14] Varma S.D., Kinoshita J.H. (1974). The absence of cataracts in mice with congenital hyperglycemia. Exp. Eye Res..

[15] Reddy G.B., Satyanarayana A., Balakrishna N., Ayyagari R., Padma M., Viswanath K., Petrash J.M. (2008). Erythrocyte aldose reductase activity and sorbitol levels in diabetic retinopathy. Mol. Vis..

[16] Li W.C., Kuszak J.R., Dunn K., Wang R.R., Ma W., Wang G.M., Spector A., Leib M., Cotliar A.M., Weiss M. (1995). Lens epithelial cell apoptosis appears to be a common cellular basis for non-congenital cataract development in humans and animals. J. Cell Biol..

[17] Chylack L.T., Wolfe J.K., Singer D.M., Leske M.C., Bullimore M.A., Bailey I.L., Friend J., McCarthy D., Wu S.Y. (1993). The lens opacities classification system iii. The longitudinal study of cataract study group. Arch. Ophthalmol..

[18] Verdaasdonk J.S., Lawrimore J., Bloom K. (2014). Determining absolute protein numbers by quantitative fluorescence microscopy. Methods Cell Biol..

[19] De Iongh R.U., Wederell E., Lovicu F.J., McAvoy J.W. (2005). Transforming growth factor-beta-induced epithelial-mesenchymal transition in the lens: A model for cataract formation. Cells Tissues Organs.

[20] Du L., Hao M., Li C., Wu W., Wang W., Ma Z., Yang T., Zhang N., Isaac A.T., Zhu X. (2017). Quercetin inhibited epithelial mesenchymal transition in diabetic rats, high-glucose-cultured lens, and sra01/04 cells through transforming growth factor-beta2/phosphoinositide 3-kinase/akt pathway. Mol. Cell Endocrinol..

[21] Balasubbu S., Sundaresan P., Rajendran A., Ramasamy K., Govindarajan G., Perumalsamy N., Hejtmancik J.F. (2010). Association analysis of nine candidate gene polymorphisms in indian patients with type 2 diabetic retinopathy. BMC Med. Genet..

[22] Taskoparan B., Seza E.G., Demirkol S., Tuncer S., Stefek M., Gure A.O., Banerjee S. (2017). Opposing roles of the aldo-keto reductases akr1b1 and akr1b10 in colorectal cancer. Cell. Oncol..

[23] Misra P., Chakrabarti R. (2007). The role of amp kinase in diabetes. Indian J. Med. Res..

[24] Kubota S., Ozawa Y., Kurihara T., Sasaki M., Yuki K., Miyake S., Noda K., Ishida S., Tsubota K. (2011). Roles of amp-activated protein kinase in diabetes-induced retinal inflammation. Investig. Ophthalmol. Vis. Sci..

[25] Mahjoub S., Masrour-Roudsari J. (2012). Role of oxidative stress in pathogenesis of metabolic syndrome. Caspian J. Intern. Med..

[26] NCD Risk Factor Collaboration (2016). Worldwide trends in diabetes since 1980: A pooled analysis of 751 population-based studies with 4.4 million participants. Lancet.

[27] Kim Y.K., Kim S.G., Kim J.Y., Heo Y.K., Park J.C., Oh J.S. (2013). Histologic evaluation of a retrieved endosseous implant: A case report. Int. J. Periodontics Restor. Dent..

[28] Kaur N., Vanita V. (2016). Association of aldose reductase gene (akr1b1) polymorphism with diabetic retinopathy. Diabetes Res. Clin. Pract..

[29] Wang Y., Luk A.O., Ng M.C., Pang C.C., Lam V., Lee S.C., Lam D.S., Choy K.W., Ma R.C., So W.Y. (2014). Additive effect of aldose reductase z-4 microsatellite polymorphism and glycaemic control on cataract development in type 2 diabetes. J. Diabetes Complicat..

[30] Tan A.G., Kifley A., Holliday E.G., Klein B.E.K., Iyengar S.K., Lee K.E., Jun G.R., Cumming R.G., Zhao W., Wong T.Y. (2018). Aldose reductase polymorphisms, fasting blood glucose, and age-related cortical cataract. Investig. Ophthalmol. Vis. Sci..

[31] Stitt A.W. (2001). Advanced glycation: An important pathological event in diabetic and age related ocular disease. Br. J. Ophthalmol..

[32] Ramana K.V. (2011). Aldose reductase: New insights for an old enzyme. Biomol. Concepts.

[33] Wautier M.P., Chappey O., Corda S., Stern D.M., Schmidt A.M., Wautier J.L. (2001). Activation of nadph oxidase by age links oxidant stress to altered gene expression via rage. Am. J. Physiol. Endocrinol. Metab..

[34] Hashim Z., Zarina S. (2011). Advanced glycation end products in diabetic and non-diabetic human subjects suffering from cataract. Age.

[35] Kumamoto Y., Takamura Y., Kubo E., Tsuzuki S., Akagi Y. (2007). Epithelial cell density in cataractous lenses of patients with diabetes: Association with erythrocyte aldose reductase. Exp. Eye Res..

[36] Wang S., Zhang M., Liang B., Xu J., Xie Z., Liu C., Viollet B., Yan D., Zou M.H. (2010). Ampkalpha2 deletion causes aberrant expression and activation of nad(p)h oxidase and consequent endothelial dysfunction in vivo: Role of 26s proteasomes. Circ. Res..

[37] Zhang C., Min Z., Liu X., Wang C., Wang Z., Shen J., Tang W., Zhang X., Liu D., Xu X. (2019). Tolrestat acts atypically as a competitive inhibitor of the thermostable aldo-keto reductase tm1743 from thermotoga maritima. FEBS Lett..

[38] Mamuya F.A., Duncan M.K. (2012). Av integrins and tgf-beta-induced emt: A circle of regulation. J. Cell. Mol. Med..

[39] Korol A., Pino G., Dwivedi D., Robertson J.V., Deschamps P.A., West-Mays J.A. (2014). Matrix metalloproteinase-9-null mice are resistant to tgf-beta-induced anterior subcapsular cataract formation. Am. J. Pathol..

